# Hemophagocytic Lymphohistiocytosis Associated with Natural T-cell Leukemia

**DOI:** 10.7759/cureus.4107

**Published:** 2019-02-20

**Authors:** Frank R Ricaurte, Tariq Kewan, Pravallika Chadalavada, Seema Misbah, Hamed Daw

**Affiliations:** 1 Hematology and Oncology, Cleveland Clinic - Fairview Hospital, Cleveland, USA; 2 Internal Medicine, Cleveland Clinic - Fairview Hospital, Cleveland, USA; 3 Oncology, Cleveland Clinic - Fairview Hospital, Cleveland, USA

**Keywords:** hemophagocytic lymphohistiocytosis, lymphoma, sepsis

## Abstract

Hemophagocytic lymphohistiocytosis (HLH) is a rare and life-threatening syndrome of excessive immune activation. It can be triggered by a variety of events that disrupt immune homeostasis, infection being the most common of them. We report a case of a 36-year-old male diagnosed with HLH associated with natural T-cell leukemia. The purpose of this report is to call attention to the clinical presentation, diagnosis, and treatment of HLH.

## Introduction

Hemophagocytic lymphohistiocytosis (HLH) occurs when macrophages and the abnormal cytotoxic function of natural killer cells become over activated, leading to a syndrome of excessive inflammation and normal tissue destruction [1]. HLH presents as a febrile illness associated with multiple organ involvement. Initial signs and symptoms of HLH can mimic common infections, fever of unknown origin, hepatitis, or encephalitis [2]. Patients with HLH can have a single episode of the disease or relapsing episodes. The initiating trigger for an acute episode is usually an infection or an alteration in immune homeostasis. HLH treatment entails a variety of options; patients who are clinically stable and have a condition responsible for triggering HLH may respond to treatment of the triggering condition alone. Unstable patients can be treated with etoposide chemotherapy, dexamethasone, bone marrow transplant, and intrathecal methotrexate for central nervous system (CNS) involvement [3].

## Case presentation

**Figure 1:**
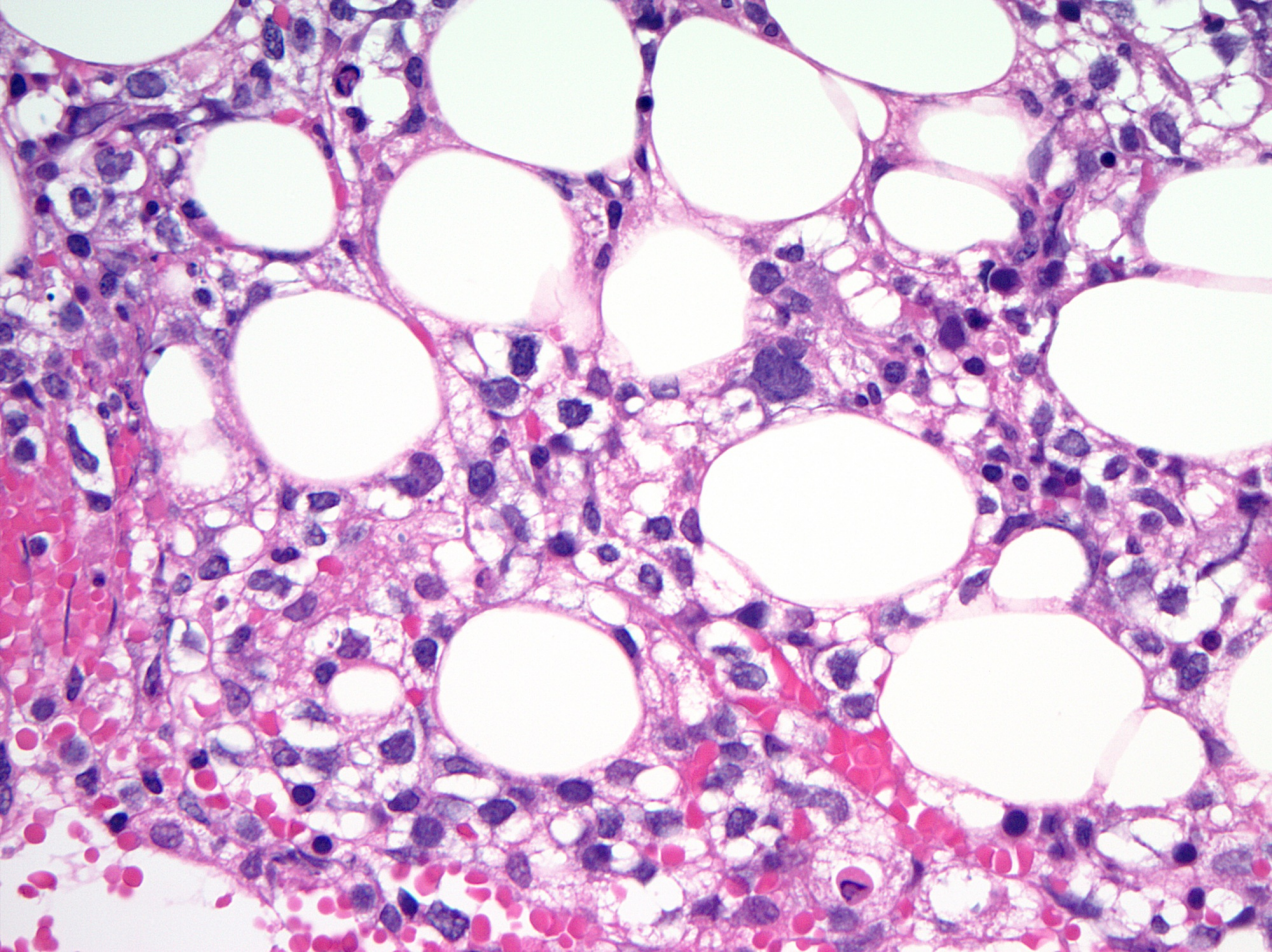
Bone marrow biopsy picture Atypical natural killer cell proliferation consistent with aggressive natural killer cell leukemia under 40x magnification.

A 36-year-old male presented to the emergency department with a chief complaint of back pain and fever. He had a past medical history of HLH diagnosed in October, 2014 and had been treated with etoposide and dexamethasone. He received four cycles of chemotherapy, and the fifth cycle was held due to chemotherapy related pancytopenia. On admission, he was febrile with a temperature up to 102°F. His absolute neutrophilic count was .03 K/uL. Magnetic resonance imaging (MRI) of the thoracic and lumbar spine was done, and no spinal or para-spinal abscess was found. The chest X-ray did not show any acute intra-pulmonary process. He was admitted for the management of neutropenic fever and was started on zosyn and vancomycin. He continued to have spikes of fever during his admission and blood cultures grew Clostridium inoculum bacteria. Also, the patient had a positive Epstein-Barr virus (EBV) deoxyribonucleic acid (DNA) test and cytomegalovirus IgG antibodies. Repeated bone marrow biopsy showed atypical natural killer cell proliferation consistent with aggressive natural killer cell leukemia, hemophagocytic macrophages, and pancytopenia (Figure 1). 

**Figure 2:**
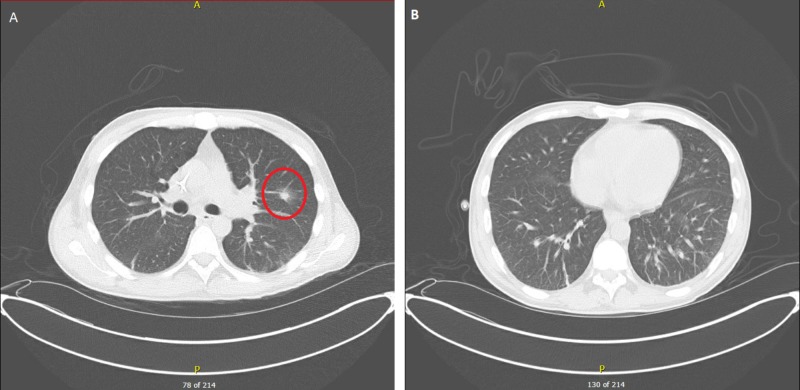
Lung computed tomography A. Red circle surrounding dominant subsolid nodule in the left upper lobe measuring approximately 10 x 8 mm with surrounding ground glass opacities; B. bibasilar reticular and ground glass opacities.

One week after admission, he started to have shortness of breath and a computed tomography (CT) scan of the chest showed new indeterminate pulmonary nodules in the left lung; the dominant nodule in the left upper lobe was measuring up to 10 mm. The nodules were more likely secondary to an infectious process including fungal pneumonia (Figure 2). The patient was started on voriconazole for a possible fungal pneumonia, prophylactic acyclovir, fluconazol, and pentamidine. Three days later, he had severe shortness of breath. Blood work-up was done and showed severe lactic acidosis and hypoxia, so he was intubated and transferred to the medical intensive care unit (MICU) for the management of septic shock. Despite aggressive management in the MICU, his acute decompensation was not prevented, and he did not respond to vasopressors. He unfortunately expired a few hours after the MICU transfer.

## Discussion

HLH cases predominantly appear in young children, but the disorder is also found in adults. Several mutations in syntaxin and perforin genes are associated with autosomal recessive HLH. Usually, childhood HLH occurs due to hereditary gene mutations. However, adult HLH cases can be triggered by a variety of cancers, immunodeficiency syndromes, and infections, especially viral ones like EBV [2].

Patients with HLH often have a diverse range of symptoms including, but not limited to, hepatomegaly, splenomegaly, lymphadenopathy, jaundice, cough, shortness of breath, diarrhea, vomiting, abdominal pain, and focal neurological symptoms. Infants with the disorder can present with irritability and abnormal growth or development [3].

Abnormally activated cells will start to attack and destroy normal body tissues and even blood cells. The persistent activation of immune system cells will lead to excessive cytokine production. It is thought that this cytokines storm is responsible for the multi-organ failure associated with HLH [4].

**Figure 3:**
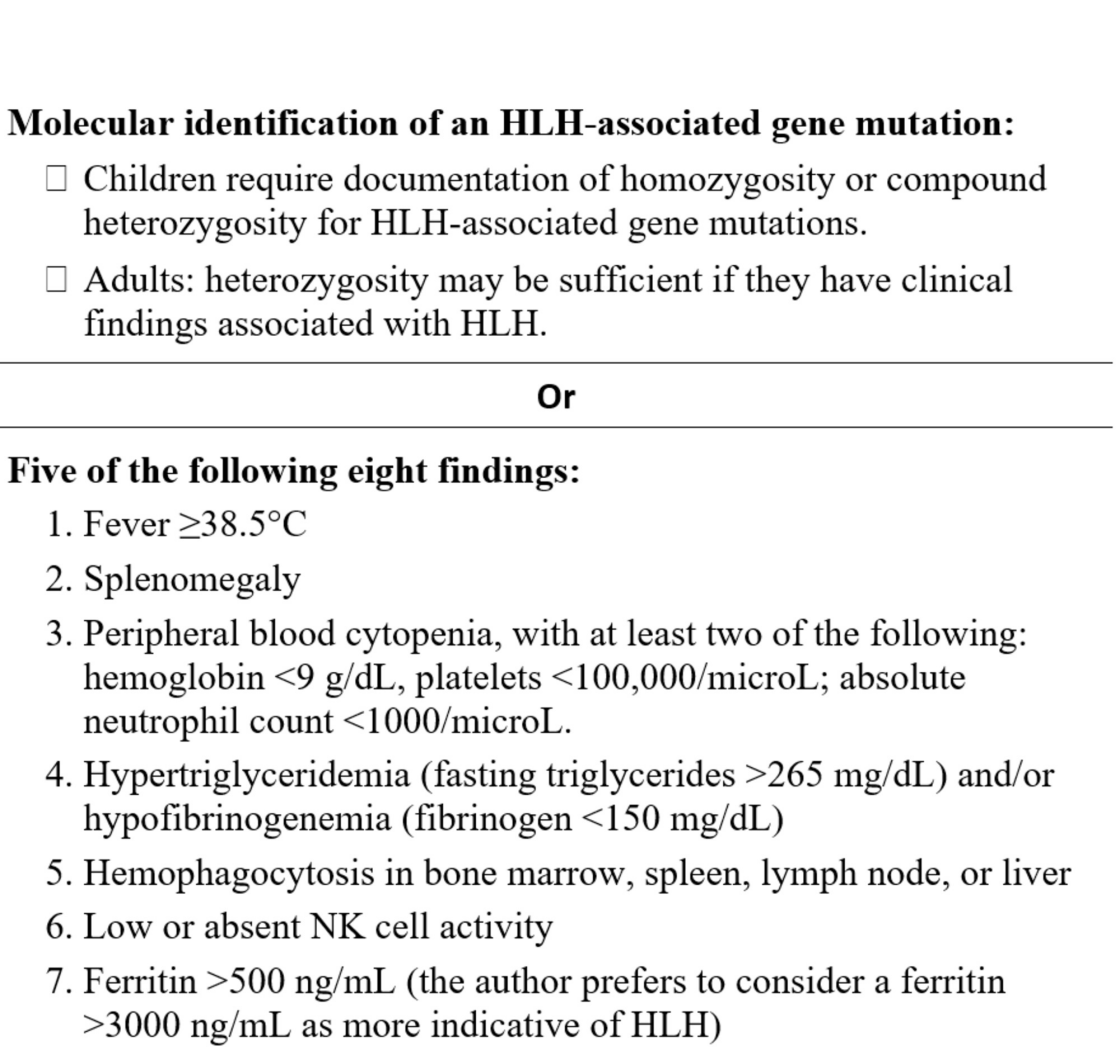
Diagnostic criteria used in the HLH-2004 trial HLH: hemophagocytic lymphohistiocytosis, NK: natural killer

HLH diagnosis is a big challenge [5]. HLH-2004 trial criteria are widely used to make the diagnosis (Figure 3). Diagnostic tests include, but are not limited to, a bone marrow biopsy, blood ferritin level, triglyceride level, soluble CD25 level, and complete blood count. Markedly elevated ferritin (>3000 ng/ml) level is considered a strong indicator of immune system activation and highly suggests HLH diagnosis [6].

Treatment for HLH calls for desperate measures considering the often-times life-threatening severity of many cases. The bulk of the treatment involves immunosuppression and attempting to reduce the amount of rapidly growing immune cells in the body. This can be achieved by chemotherapy, steroids, and anti-thymocyte globulin (ATG) treatment. Bone marrow stem cell transplants are also on the treatment spectrum for completely curing the disorder. Therapy based on the HLH-94 protocol consists of eight weeks of induction therapy with etoposide and dexamethasone, with intrathecal therapy for those with central nervous system (CNS) involvement [7-8]. Fortunately, a new drug, Gamifant, has been approved for use on primary HLH with refractory, recurrent, or progressive disease. It's a monoclonal antibody that binds and neutralizes interferon gamma [9].

In our case, the patient received four cycles of HLH-94 protocol chemotherapy and developed pancytopenia secondary to the treatment. He presented with neutropenic fever and sepsis secondary to Clostridium inoculum bacteremia that primary responded to zosyn, given his negative second set of blood cultures. However, he was severely immunocompromised and had possible fungal pneumonia that did not respond to voriconazole treatment. He had a severe septic shock that did not respond to supportive MICU treatment. 

In other ongoing clinical trials of HLH, there is a multitude of cases that have commonalities regarding specific genetic mutations. In many cases of primary HLH, the disorder has shown to have a shared cause of FLH loci mutations [10]. The treatment of other ongoing clinical trials of HLH slightly differs from the treatment and management in this case. With early diagnoses, numerous patients received hematopoietic stem cell transplantation, which predominantly led to remission [10].

## Conclusions

This case emphasizes the proper tests that should be taken to make a correct HLH diagnosis as well as the appropriate treatment options, which again include chemotherapy, steroids, immunotherapy, and bone marrow stem cell transplants. These options should be used, of course, in HLH patients depending on the severity and characteristics of each individual case.

## References

[1] Tsuda H (2018). Hemophagocytic syndrome (HPS) in children and adults. Int J Hematol.

[2] Sullivan KE, Delaat CA, Douglas SD, Filipovich AH (2018). Defective natural killer cell function in patients with hemophagocytic lymphohistiocytosis and in first degree relatives. Pediatr Res.

[3] George M (2018). Hemophagocytic lymphohistiocytosis: review of etiologies and management. J Blood Med.

[4] Risma KA, Marsh RA (2018). Hemophagocytic lymphohistiocytosis: clinical presentations and diagnosis. J Allergy Clin Immunol Pract.

[5] Jordan MB, Allen CE, Weitzman S, Filipovich AH, McClain KL (2018). How I treat hemophagocytic lymphohistiocytosis. Blood.

[6] Hindi Z, Khaled AA, Abushahin A (2018). Hemophagocytic syndrome masquerading as septic shock: an approach to such dilemma. SAGE Open Med Case Rep.

[7] Wong ET (2018). Management of central nervous system lymphomas using monoclonal antibodies: challenges and opportunities. Clin Cancer Res.

[8] Henter JI, Aricò M, Egeler RM (1997). HLH-94: a treatment protocol for hemophagocytic lymphohistiocytosis. Med Pediatr Oncol.

[9] Al-Salama ZT (2018). Emapalumab: first global approval. Drugs.

[10] Elyamany G, Alzahrani A, Elfaraidi H, Alsuhaibani O, Othman N, Mussaed EA, Alabbas F (2016). Hemophagocytic lymphohistiocytosis: single-center series of 12 cases from Saudi Arabia. Clin Med Insights Pediatr.

